# Recent Advances in Droplet-based Microfluidic Technologies for Biochemistry and Molecular Biology

**DOI:** 10.3390/mi10060412

**Published:** 2019-06-20

**Authors:** Joel Sánchez Barea, Juhwa Lee, Dong-Ku Kang

**Affiliations:** 1 Department of Chemistry, Incheon National University, Incheon 22012, Korea; joesbarea@inu.ac.kr (J.S.B.); juhwalee94@inu.ac.kr (J.L.); 2 Department of Chemistry, Research Institute of Basic Sciences, Incheon National University, Incheon 22012, Korea

**Keywords:** droplet-based microfluidic, biochemistry, molecular biology, digital PCR, biochip, biosensor, digital quantification, microfluidic, single cell analysis

## Abstract

Recently, droplet-based microfluidic systems have been widely used in various biochemical and molecular biological assays. Since this platform technique allows manipulation of large amounts of data and also provides absolute accuracy in comparison to conventional bioanalytical approaches, over the last decade a range of basic biochemical and molecular biological operations have been transferred to drop-based microfluidic formats. In this review, we introduce recent advances and examples of droplet-based microfluidic techniques that have been applied in biochemistry and molecular biology research including genomics, proteomics and cellomics. Their advantages and weaknesses in various applications are also comprehensively discussed here. The purpose of this review is to provide a new point of view and current status in droplet-based microfluidics to biochemists and molecular biologists. We hope that this review will accelerate communications between researchers who are working in droplet-based microfluidics, biochemistry and molecular biology.

## 1. Introduction: Biochemistry and Molecular Biology for Droplet-based Microfluidics

In the past three decades, biochemistry and molecular biology have been developed in various basic research areas and applications in which microfluidics have played one of the most common roles [1,2]. It has been a key goal to search for high throughput results while reducing the amount of sample and reagents required for the experiment, and to increase the sensitivity and precision of the assay over the last decade. However conventional experimental approaches suffer from their limitations in terms of low throughput, large sample consumption and false positive (or negative) errors from the assays with complex biological samples.

**Figure 1:**
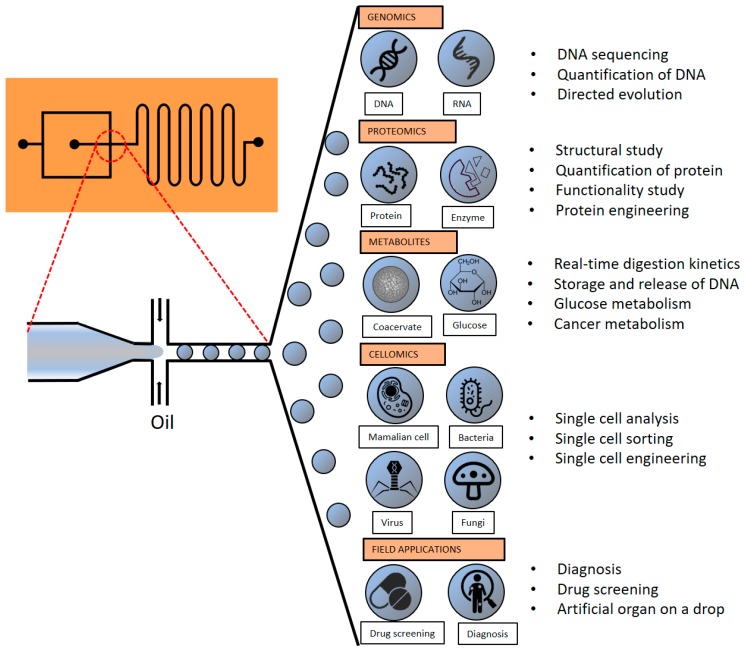
Schematic diagram of biological components that can be analyzed and engineered using droplet-based microfluidics (DMFs).

In recent years, droplet-based microfluidic (DMF) technologies have been focused on reducing fluids to the nano-to-femtoliter scale. This reduction makes the flow in the channels become laminar, represented by a low-Reynolds number, which gives derivate properties like the immiscibility of the different phases of the fluid. It changes the fluid’s properties as the flow in the channels will be laminar, represented by a low-Reynolds number, which gives derivate properties like the immiscibility of the different phases of the fluid. Under the aforementioned conditions the samples are divided into droplets for better manipulation of the properties of the assays [3]. To prevent the coalescence of the droplets, surfactants is important depending on the goal of the assay. In particular, there is a great variety of different surfactants for hydrocarbon oil or silicon oil [4]. The techniques derived from this field have emerged as potential high throughput platforms for performing various biochemical and molecular biological experiments. The term DMF sometimes can make reference to digital microfluidics which not only includes systems based on droplets in microchannels but also to electrowetting on dielectric (EWOD) referring to those microfluidic devices that manipulate the droplets by electric signals [5]. In this review, we will focus only on droplet-based microfluidics without further details on EWOD. DMF systems provide tools to precisely manipulate and simultaneously analyze single biological molecules within femto-to-nanoliter droplets in an ultrahigh throughput manner (Figure 1). Compartmentalization of biological molecules within microdroplets provides numerous advantages over conventional approaches, in terms of higher target concentration and also low number of background molecules [6]. These advantages reduce the reaction time, and increase the sensitivity and selectivity that enable low false positive and false negative rates [7]. DMF systems also offer the reduction of equipment, portability and facile integration of functional components [2,8,9]. 

Genes, proteins and cells are fundamental building blocks of life and have been tremendously studied in biochemistry and molecular biology to understand the mechanism of life. In this review, we briefly introduce selected recent advances of DMF technologies for biochemistry and molecular biology, with particular emphasis on genomics, proteomics and cellomics, then highlighting also the possibilities in biological applications.

## 2. Droplet-based Microfluidics for Genomics

Complete genomic studies of organisms in all other respects, including whole genome sequencing, directed evolution, quantification of DNA/RNA, and even epistatic, pleiotropic and heteroidal interactions of genes, are the most important in understanding the function of genes in biochemistry and molecular biology fields. In recent years, DMF technologies have been merged with molecular biology approaches for the understanding and also for engineering genetic information through whole genome sequencing and quantification of nucleic acids [10]. This special interest has enabled advances in amplification and analysis of DNA and RNA within microdroplets, as well directed evolution approaches, and have found a great tool to improve their efficiency. Very recently, DMF also applied the study of multiplex chromatin-interaction analysis based on single-molecule precision chromatin interactions that can provide topological insight into transcription [11]. 

### 2.1. DNA Sequencing

**Figure 2:**
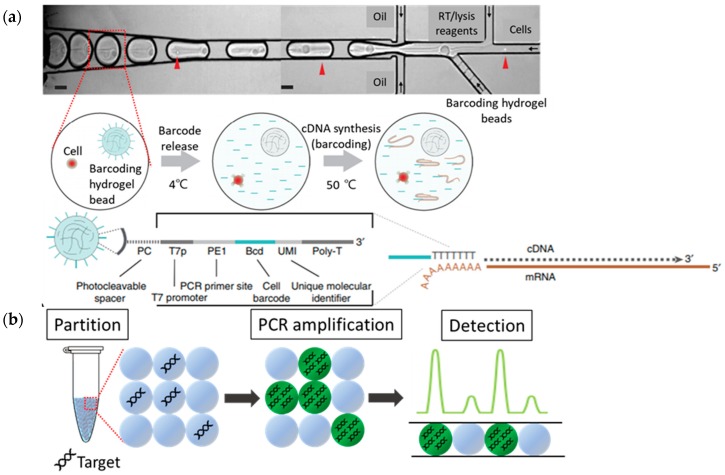
(**a**) DMF-based single cell transcriptome barcoding and genomic sequencing. in drops, reproduced with permission from [18], published by Springer Nature, 2016. (**b**) Digital PCR for the absolute quantification of target DNA. Target sequences are encapsulated and amplified in pico to nanoliter droplets. Then, a number of target molecules can be quantified by counting fluorescent droplets.

Among the different techniques used in genomics, DNA sequencing is probably one of the most important tools for understanding genetic information. DMF technologies have advantages in terms of throughput, sample amount and assay time for the identification of DNA and also for the single cell-based study. Single-molecule droplet barcoding (SMDB) is one of excellent examples of an ultrahigh-throughput method to barcode long DNA molecules for short-read sequencing [12]. Using DMFs, barcoded-single DNA molecules are isolated in aqueous droplets by one million times smaller than conventional well plates. The Single Cell genome-sequencing (SiC-seq) technology is used for the encapsulation of a single bacterial cell within micro-sized hydrogels to lyse bacteria and merge them individually with a barcode for template fragmentation and barcoding [12,13]. Theoretically, all sizes can be amplified, but inefficiencies have been reported with templates over 3 kb. Another example for DNA sequencing is the use of a Förster resonance energy transfer (FRET) ligation assay with probes targeting different sequences for detecting polymorphisms of single-base mismatch discrimination [14]. Kogawa and colleagues recently introduced a new analytical tool named “Cleaning and Co-assembly of a Single-Cell Amplified Genome (ccSAG)” that combined with parallel single-cell genome amplification obtains composite single-cell genomes. In this approach, multiple single-cell amplified genomes (SAGs) can be obtained with low contamination, (less than 1.25%) from the bulk DNA of single gut microbial cells [15]. Petukhov and co-workers also developed a single-cell RNA sequencing protocol (dropEst) using DMFs that can provide accurate estimates of molecular counts in individual cells by correcting sequencing errors affecting molecular and cellular barcodes [16]. Single cell sequencing has an increasing importance in biotech procedures related to diagnosis and personalized medication [17]. Recently, throughput of DMF-based single cell sequencing was achieved single cell RNA sequencing from 15,000 cells within an hour (Figure 2a) [18]. Zilionis and co-workers introduced the inDrops methods which has the capability of analyzing the mRNA transcriptome from thousands of individual cells by single-cell encapsulation with barcode-conjugated hydrogel beads. Antibiotic-resistance genes, virulence factors and phage sequences were also determined from a single-cell for analysis of the microbial communities distribution, present in environmental samples [19,20]. More recently, Taqman PCR and molecular beacons were merged into DMF technologies to validate single nucleotide polymorphism (SNP) and it provides a single cell-based genotyping approach [21,22].

### 2.2. Identification and Digital Quantification of Target DNA

PCR (polymerase chain reaction) technology has been evolved with the use of DMF technologies for the precise detection and absolute quantification of the nucleic acid of interest (Figure 2b). Basically, single-target DNA or target RNA could be encapsulated within nanoliter to picoliter droplets with low-copy numbers of background DNA (or RNA). The advantage of DMF-based digital PCR is the small reaction volume that provides (1) higher target concentration per reaction (microdroplet), (2) accumulation of amplified signal within microdroplets and (3) less background molecules by partitioning large volumes of sample into nanoliter to picoliter droplets. The other advantage of DMF-based digital PCR is that it does not require normalization using a standard curve because a digital PCR reaction is basically targeting single copy of DNA within microdroplets and it provides absolute quantification.

Zhao and co-workers recently introduced oil-saturated polydimethylsiloxane (PDMS) chip (OSP) for the on-chip droplet digital PCR (ddPCR) that was used for absolute quantification of lung cancer related microRNA and pathogenic bacterial DNA [23,24]. Eastburn and co-workers used ddPCR for cell-type identification among a heterogeneous population of mammalian cells based on gene expression [25]. In their approach, reverse transcription PCR and DMF technology were integrated for monitoring prostate cancer-related RNA from single-cell encapsulated droplets. ddPCR has been proven useful with numerous examples in the detection of circulating tumor DNA (ctDNA) in blood, urine and also solid tumor samples [26,27,28,29,30,31,32,33,34,35,36,37,38,39,40,41]. Joensson and coworkers integrated ddPCR with fluorescent color-coded Luminex®beads for multiplex detection of pathogen DNA biomarkers [42]. Droplets were generated with three different Luminex bead sets coupled to target-specific capture oligos to detect the three microorganisms infecting poultry (avian influenza, infectious laryngotracheitis virus and *Campylobacter jejuni*) through hybridization with a DNA mixture containing pathogenic DNA. Then, target DNA was amplified with fluorescently labeled target-specific primer within picoliter droplets and Luminex bead were collected for the analysis in the Luminex instrument.

#### Isothermal Amplification

**Figure 3:**
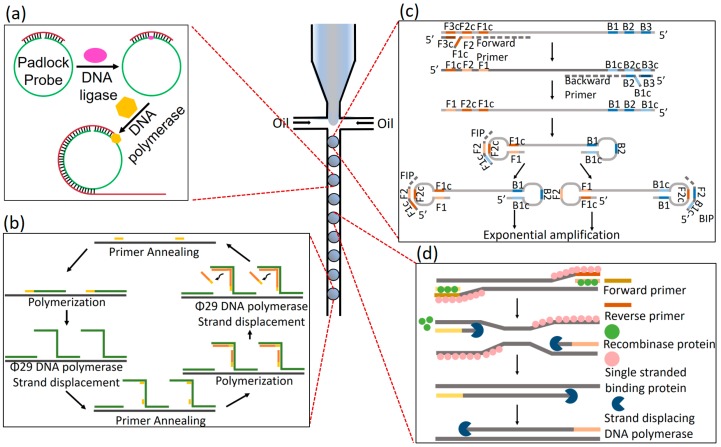
Schematic of isothermal amplification in a droplet. (**a**) Rolling circle amplification (RCA). (**b**) Isothermal multiple displacement amplification (IMDA). (**c**) Loop-mediated isothermal amplification (LAMP). (**d**) Recombinase polymerase amplification (RPA).

Nucleic acid amplification is a key procedure for the detection and quantification of biomarker nucleic acids. Conventional PCR amplification requires repeated cycles of three or two temperature-dependent steps during the amplification processes which requires a thermocycler. In contrast, isothermal amplification methods have no need for thermal cycling that can be used in simplified-microfluidic systems including DMF-based ddPCR. Various isothermal amplification methods have been developed and merged in DMF for precise detection and absolute quantification of the nucleic acid of interest (Figure 3).

Rolling circle amplification (RCA) consists of an isothermal enzymatic process where a long single stranded DNA or RNA is formed using a circular template for the amplification of a short primer (Figure 3a). The product of RCA is a concatemer which contains tens to hundreds of pair repeats that are complementary to the circular template [43]. Knudsen and co-workers demonstrated detection of malaria (*Plasmodium*) in blood and saliva by integration of DMF with RCA [44]. Leong and coworkers also applied RCA in DMF for the monitoring *Plasmodium* topoisomerase I (pTopI) in the saliva of the patient [45]. 

Multiple displacement amplification (MDA) allows massive parallel amplification of single cell genomes while maintaining the accuracy and specificity of the sequence (Figure 3b). Takeyama and co-workers introduced whole genome amplification (WGA) for single-cell sequencing using DMF [46]. Tens of thousands of single cells were individually encapsulated in millions of picoliter droplets and then subjected to lysis and WGA in DMF. This approach enables single-cell based high-throughput acquisition with contamination-free sequencing that results in 21,000 single-cell sequencing within an hour. Another group presented a droplet digital MDA (ddMDA) technique where the division of the DNA template into thousands of sub-nanoliter droplets reduces the competition among DNA fragments for primers and polymerase, greatly reducing amplification bias [47]. 

Loop-mediated isothermal amplification (LAMP) uses multiple primer sets for a single nucleic acid target. It consists of an auto-cycling strand displacement DNA synthesis that is catalyzed by DNA polymerase with high strand displacement (Figure 3c) [48]. Rane and colleagues recently reported an integrated-microfluidic system for digital nucleic acid detection through droplet generation, incubation and in-line detection for digital LAMP within a single device [49]. Also, a hydrophilic PDMS that allows LAMP to be performed in a self-driven DMF device has been announced [50]. For the detection of influenza A virus [51] and Zika virus [52], reverse transcriptase loop-mediated isothermal amplification (RT-LAMP) has also been merged with DMF technologies. 

Continuous-flow digital LAMP is another innovation for the integration of this amplification technique with droplet-microfluidics, which has been already utilized for ultrasensitive DNA detection [49]. The group of Giuffrida and colleagues described a combined method for detecting microRNA-210 sequences using digital microfluidics and molecular-beacon (MB)-assisted isothermal circular-strand-displacement polymerization (ICSDP) [53]. The same group also show a new method for the detection of nanoliter droplets of nucleic acids, specially microRNA sequences in a picomolar scale [54]. 

Helicase dependent amplification (HAD) consist of using a probe with a hairpin structure that bears the transcription factor binding site to convert the protein signal to the DNA signal. Cao and colleagues demonstrated a simple method for the detection of a transcription factor. In the absence of a transcription factor zero background signal can be achieved due to the digestion of excess probes by the exonucleases and the subsequent one primer-triggered high fidelity amplification [55]. 

Recombinase-polymerase amplification (RPA) uses two oligonucleotide primers specific to the target, which binds to the template DNA assisted by a recombinase in combination with strand-displacement DNA synthesis (Figure 3d). Recently, this method has been integrated into DMF technologies [56] including Slip chip [57], centrifugal step emulsification droplets [58] and a chip-based picoliter well array [59]. 

## 3. Droplet-based Microfluidics for Proteomic

**Figure 4:**
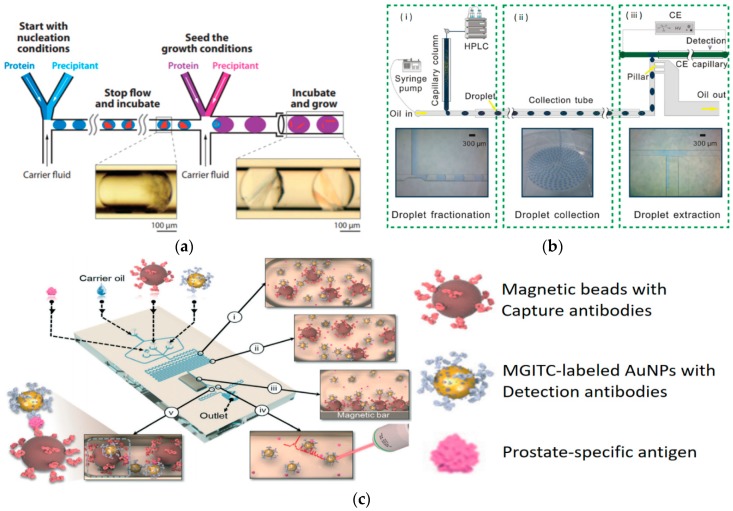

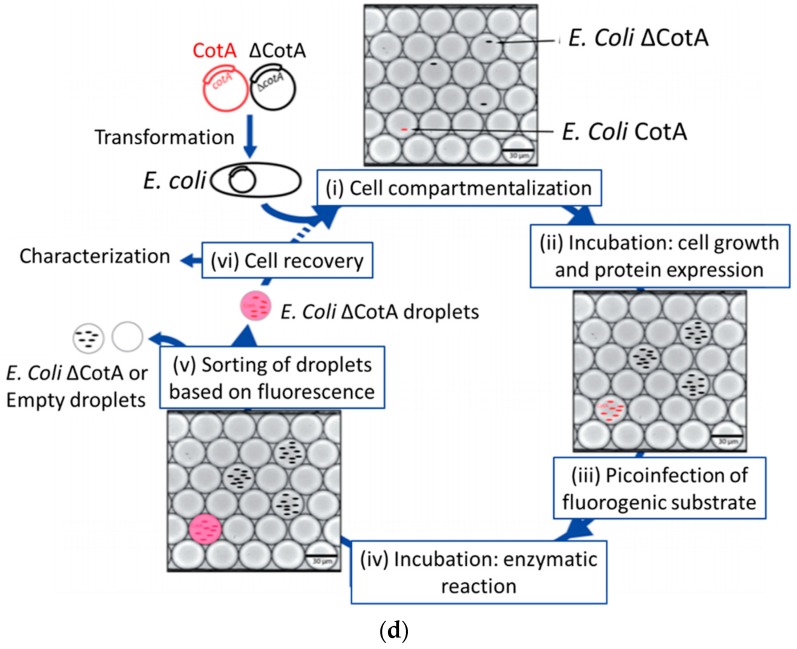
Droplet-based microfluidics for proteomics. (**a**) Separating the stages of nucleation and growth in the crystallization of “severe acute respiratory syndrome (SARS) protein” yields single crystals, reproduced with permission from [62], published by John Wiley and Sons, 2006. (**b**) A droplet-interfaced 2D nano liquid chromatography-capillary electrophoresis (LC-CE) system. The HPLC effluent is fractionated into a series of nanoliter droplet units right after chromatography (panel (i)), and collected and stored in a tube (panel (ii), tube inside diameter: 0.38 mm), before drop-wise analyzed in capillary electrophoresis (CE) (panel iii), reproduced with permission from [72], published by Elsevier, 2015. (**c**) Schematic illustration of the surface enhanced raman scattering (SERS)-based microdroplet sensor for wash-free magnetic immunoassay, reproduced with permission from [77], published by Royal Society of Chemistry, 2016. (**d**) A flexible droplet-based micro-fluidic platform that can be used for high-throughput screening of *Escherichia coli* cells for measuring the CotA enzymatic activity, reproduced with permission from [84], published by Royal Society of Chemistry, 2014.

Understanding proteome, structure and function of proteins in tissues is the next generation challenge in molecular biology. In particular, throughput is one of the important factors in the study of protein expression and its structure, and this is due to the vast amount of data from various tissues and cells [60]. Recently, DMF technology has been applied for proteomics and also for the identification of protein structure and its function [61]. Diversity of assays in the field of proteomics require the control of a large number of parameters due to the complexity of the samples, and since a wide variety of proteins can be found in the small amount of sample, there is a necessity of precise methods of analysis. In particular, protein crystallization requires exquisite control of the parameter and DMF technology integrated here for precise manipulation of complex procedures. Within a single microfluidic device, multiple functions such as separation of the nucleation from growth stages by stopping the flow and combination of high-concentration protein with precipitant solutions to form droplets are included. Then, the flow stops to proceed with the incubation to generate seed crystals. When the precipitant solutions are combined with lower-concentration protein droplets containing lower supersaturated solutions are formed to produce crystal growing, then each growth droplet that contains seed crystals flows through a glass capillary and is incubated (Figure 4a) [62]. In recent years, it has been seen how DMF technology provide new perspectives and solutions in these different fields of proteomics but there are still many challenges such as the implementation in point of care devices. 

### 3.1. Identification and Structure of Protein

The structure of proteins has been studied in molecular biology and biochemistry for a long time and even nowadays it is one of the most common topics in this field. Protein crystallization at a droplet scale allows the use of reagents and protein samples to be minimized which contributes to reducing the cost of the process and at the same time to controlling the diffusion of molecules and the nucleation of crystals [63,64,65]. Maeki and colleagues suggested that the droplet size and the diffusion are the most common factors for the nucleation of proteins [66]. For evaluating protein crystallization conditions, another group presented a droplet-based composite PDMS/glass capillary microfluidics system using microbatch and vapor-diffusion methods with on-chip X-ray diffraction [67]. The use of picoinjection and Venturi junction can generate droplets of supersaturated solution to eliminate the problem of unspecific precipitation [68].

Protein quantification methods increased their efficiency and sensitivity using DMF as it has been demonstrated with the development of digital amyloid quantitative assay (d-AQuA). The d-AQuA uses DMFs for absolute quantification of single insulin paragons at a single molecule sensitivity with a phase of amplification by serial dilutions. This approach is being possible to be used for the monitoring of protein misfolding and aggregation (PMA) diseases [69]. Quantitative characterization at the single cell level can be a challenge due to the small amount of target protein within complex samples and a DMF-based barcoding approach allows cells to be distinguished by the protein expression profile, with limitless multiplexing [70]. More recently, advanced-multiplexing detection methods have been shown based on DMF-based up-conversion nanoparticles (UCNPs) [71]. In other detection and quantification methods such as chromatography of complex mixtures, one of the issues is a coelution due to their complexity and peak dilution is also inevitable because of molecular diffusion during sample transfer. It causes the remix of separated peaks and band broadening, which produces the degradation of achievable resolution. To address this issue, DMF technology has been integrated with liquid chromatography and capillary electrophoresis [72]. In this droplet-interface 2D system, the HPLC effluent is fractionated into nanoliter droplets and collected in a tube right after performing the chromatography and before proceeding to the analysis of the drops (Figure 4b). 

### 3.2. Functionality Study

Protein–protein interaction studies, immunoassays and enzymatic assays are the main examples of how DMF technologies have been utilized to determine the functions of proteins. Enzymes are challenging to engineer due to the complexity of relationship between the sequence and biochemical properties. DMF technology can provide ultrahigh-throughput methods for mapping the enzyme sequence–function relationship by combining droplet microfluidic screening with next-generation DNA sequencing [73].

Immunoassays are one of the most common tools in molecular biology and biochemistry, for identification and quantification of different pathogens and markers in biological samples which makes it a common assay for diagnosis. DMF technologies can provide new advances in this technique like avoiding the washing steps at same time as reducing the time of the assay and the sensitivity, as proven in the microfluidic bead based immunoassay detecting even 0.01µg mL^−1^ of C-reactive proteins [74]. Performing immunoassays like ELISA for the detection of single exosomes can be a very useful tool for cancer diagnosis, and combined with DMF technology, it can encapsulate 1 exosome per droplet requiring a very low concentration of exosome in the samples [75]. An automated DMF device for high temporal resolution studies on hormone secretion has also been developed; this device performs fluorescence imaging at same time the droplets are forming [76]. The use of surface enhanced raman scattering (SERS) with droplet microfluidics can also be applicable to the detection of biomarkers like prostate specific antigen (PSA). The combination of four compartments for generating microdroplets, separating immunocomplexes droplets and another for collecting unbound SERS nanotag droplets for detection allows the immunoassay for PSA to be performed. It contains five compartments including 1) droplet generator, where magnetic immunocomplexes are formed and 2) droplet sorting with magnetic field. Following this, 3) larger droplets are generated containing the supernatant for SERS detection and 4) unbound nanotags are separated by droplet fission from the small droplets containing the sandwich immunocomplex. 5) The SERS intensity is measured in both droplet cases showing high signal for the immunocomplex compared to the low signal of the unbound nanotags (Figure 4c) [77].

The activity of enzymes as well as their structure are fields of study that require control of the environment; DMF allows the control of all the different parameters and the study of various classes of enzymes like hydrolases, aldolases, proteases, amino-acid dehydrogenases and polymerases [78]. Also, cell-secreted enzymes have been used to demonstrate a high-throughput screening method combining optical and fluorescence microscopy with MALDI-MS in a single platform. It has a potential to quantify and characterize the enzymes that secreted from single cell but it also required creating small-size droplets for single cell-based absolute quantification of enzyme [79]. The use of hydrogel immunosensors is another approach for single-cell multiplexed secretion analysis that has been proven recently [80]. The main approach to study enzymes is probably directed evolution assays.

Directed evolution allows proteins and RNA with properties which are not found in nature due to random mutations of the genes that encode the targeted protein to be obtained. A good example of the application of DMF technology to directed evolution, is the absorbance activated droplet sorter (AADS) that sorts microdroplets based on an absorbance readout up to 300 droplets per second. The efficiency of AADS on protein engineering was proven by improving the activity of phenylalanine dehydrogenase toward its native substrate after two rounds of directed evolution [81]. Enzymatic properties such as chemospecificity and enantiospecificity can be generated within droplet and it can be manipulated using a Droplet Microfluidics Droplet Sorter (DMDS) with a dual channel fluorescence–activated droplet sorting (DC-FADS). This is an effective method that can be useful for drug development. After several rounds of mutagenesis and screening, specific enantiomers can be selected [82]. For fluorescence based assays, hydrophilic rhodamine-based enzymatic substrates have been used in DMF systems that can prevent the chromophore transfer between droplets, making the sorting of the droplets due to their fluorescence signal more effective [83]. The picoinjection of the fluorogenic substrate to the droplets to modify the incubation period depending on the kinetics of the enzyme of study allows the analysis of 10^6^ variants in one hour. This screening platform consists of different steps, starting with the encapsulation of *Escherichia coli* previously transformed with CotA (active enzyme) and ΔCotA (frameshifted enzyme) in different strains, with induction medium. In the next step, the emulsion is incubated off-chip to induce the expression of the different enzymes within the droplets. After the incubation step, fluorogenic substrate was introduced into pre-generated droplets with a picoinjection microchannel and droplets were off-chip incubated for the enzymatic reaction. Then, florescent droplets were sorted out to isolate target-cells based on the enzymatic activity and they were recovered from the sorted droplets by droplet breakage reaction. Recovered cells can be used for determining the enrichment with the active enzyme or follow another round of selection (Figure 4d) [84].

### 3.3. Protein Engineering and Directed Evolution

Protein engineering is the process of developing polypeptides or proteins by modification of amino acid sequences that are found in nature, and have become a common tool for the development of proteins applied to industry and the clinic [85]. Combining DMF systems with other techniques such as mass spectrometry provides great improvement in protein engineering [86]. Directed evolution has become one of the most common strategies of protein engineering to improve valuable proteins for various bio-industries including bioenergy, biomass-processing and bio-sustainability [87]. Merging DMF systems with directed evolution assays reduces the volume of sample needed from 100 µL to 6 pL, enabling the analysis of more than 1 × 10^8^ reactions/day compared to the 73,000 reactions per day that can be analyzed with previous technologies [81]. However, success of this approach depends on a diversity of combinational libraries and it requires an efficient method for the high-throughput screening approach for large libraries of variants [88]. DMF technology has the ability to generate uniform droplets and is also suitable for encapsulating single cells or for in vitro expression of single genes within pico to nanoliter water-in-oil droplets [89]. Due to the uniformity and tiny size of droplets, this approach provides quantitative assays in a high-throughput manner and another advantage is the ability for sorting target droplets simultaneous and continuously in real-time. Other groups have presented droplet-based optical polymerase sorting (DrOPS) as a general strategy for expanding the polymerase function by employing an optical sensor to monitor the activity of the polymerase inside the microenvironment of a uniform synthetic compartment generated by microfluidics to engineer natural polymerase to replicate unnatural genetic polymers. Hosokawa and colleagues used agarose gel based DMF technology for the screening of lipolytic enzyme genes from the metagenomic library constructed from soil by *E. coli* that is transformed with a metagenome [90].

## 4. Droplet-based Microfluidics for the Study of Other Metabolites

Recently, awareness of the importance of lipids has been increasing. In particular, studies on structure and roles of the lipidome have been diverse in biochemistry and molecular biology. The relationship between structure and behavior of membranes is a traditional research topic in molecular biology and also one of the most popular topics in biotechnology because the mimic of membranes is playing a new interesting role. DMF technology has recently become a tool to precisely manipulate lipid-based membranes for various approaches.

**Figure 5:**
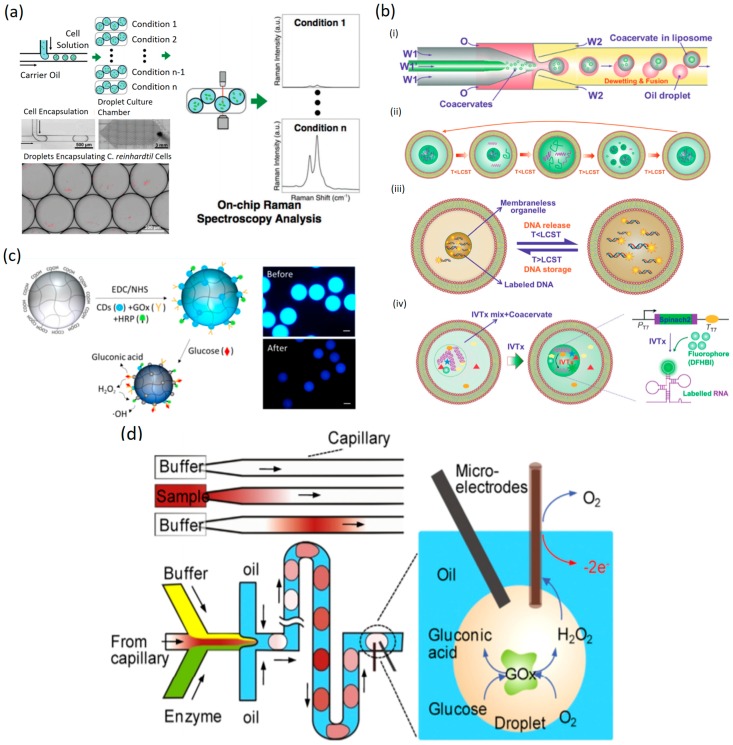
Droplet-based microfluidics for metabolites. (**a**) Overview of Raman spectroscopy integrated with a polydimethylsiloxane (PDMS) droplet microfluidic platform for on-chip droplet formation, culture and in vivo cellular lipid analysis, reproduced with permission from [92], published by Royal Society of Chemistry, 2017. (**b**) Encapsulation of coacervates into liposomes. The microfluidic preparation of water/oil/water (W/O/W) double emulsions with coacervates, reproduced with permission from [94], published by Wiley-VCH Verlag GmbH and Co. KGaA, 2017. (**c**) The prepared fluorescent hydrogel glucose biosensor (FHGB) droplets showed a dual response to glucose of carbon dot fluorescence quenching and droplet size reduction upon bienzymatic reaction with glucose, reproduced with permission from [98], published by American Chemical Society, 2018. (**d**) Schematic illustration of the principle of concentration gradient generation, the sensor is based on the electrochemical detection of glucose in a droplet, reproduced with permission from [99], published by Elsevier, 2014.

DMF allows the precise control of the environment where the planar lipid bilayer membranes are placed which facilitates the study of their properties [91]. The production of lipids by microalgae can be measured by combining DMF technology with Raman spectroscopy to characterize microalgae lipid production in a PDMS device with a method using FC-40 carrier oil to overcome the high Raman background of PDMS (Figure 5a) [92].

Another type of study performed recently was the real-time digestion kinetics based on triglyceride droplets that contained various lipophilic micronutrients. Whilst being monitored with a confocal fluorescence microscope, oil droplets were generated and trapped in the PDMS chip, facilitated by the hydrophilic property of the PDMS. This experiment was focused on the digestion of beta-carotene and observing its degradation, but the method can be applied to various studies related to toxicology, pharmacology and nutrition [93]. The study was extended to other oils and pro-vitamins as well as the effect of the gastric phase of digestion on the intestinal phase [93].

Coacervates are spheres formed by liquid–liquid phases divided by membranes. Coacervates have been studied for a few decades and a new strategy has been presented to construct single monodisperse coacervate using liposome-microdroplets via a bottom-up approach to construct protocells. In this approach, DNA is sorted and released from the encapsulated coacervates as well as localized transcription. The encapsulation is produced by fusion of small coacervates into big coacervates in liposomes and then the release or storage of the DNA within liposome droplets can be analyzed over time depending on the temperature changes. DNA was stored at a higher temperature and then transcription was monitored with a Spinach 2 aptamer and 3,5-difluoro-4-hydroxybenzylidene imidazolinone (DFHBI) (Figure 5b) [94]. Wang and colleagues developed a polyethyleneglycol (PEG)-based triblock copolymers to assemble coacervate in a DMF-based noncovalent method [95].

The study of carbohydrates with DMF technology is focused mainly on glucose metabolism related to cancer and the lactate release. Multiplexing single-cell measurements of the uptake and release of carbohydrates by cancer cells can be of great importance to help better understand cancer metabolism [96,97], but also the measurement of glucose is a topic widely studied with DMF technology. One of the examples is of monitoring glucose using a microhydrogel biosensor. This technique is based on the bi-enzymatic reaction of glucose oxidase (GOx) and horseradish peroxidase (HRP). The gluconic acid is generated by GOx and it causes the quenching of carbon dots. The size reduction of carbon dots is induced by the –OH radicals that are produced by HRP (Figure 5c). The electrochemical detection approach also can be used to measure glucose concentration by coupling with DMF technology and GOx produced H_2_O_2_ that provides −2e^−^ (Figure 5d) [98,99,100,101,102].

## 5. Droplet-based Microfluidics for Cellomics

The study of cells and their properties as well as their interactions requires a controlled environment with appropriate conditions for the growth, culture or assembly of artificial cells. Detection of single cells can be a powerful tool for diagnosis of various kinds of diseases. DMF technologies have emerged as a great solution for various studies in cellomics including single cell proteomics and single cell engineering. DMF technology improves the performance of single cell analysis and sorting among other applications increasing the amount of cells that can be analyzed from 1536 cells in one assay to more than 300 droplets per second, which greatly increases the throughput of the experiments [103]. 

### 5.1. Single Cell Analysis

Precision in characterization and in the study of biochemical reactions and interaction of cells is essential to every analysis in cellomics. DMF technology provides a cell-to-droplet approach that increases the sensitivity of single cell phenotype analysis, as well as more specific analysis like the specific binding of antibodies to target cells [104]. A common tool for the analysis of single cells is the implementation of SERS on droplet microfluidics. Multiple examples have been demonstrated like the detection of PC3 cells in a well for droplet interrogation after encapsulating these cancer cells with wheat germ agglutinin (WGA) [105]. The detection of various bacteria and eukaryotic cell lysate have also been reported [106,107,108]. For the identification of leukemia cell lysate, a new approach was presented which uses SERS-active silver nanoparticles and a droplet-based microfluidic chip. Injecting silver nanoparticles, KCl as aggregation agent and cell lysate containing cell constituents, such as nucleic acids, carbohydrates, metabolites and proteins into a continuous flow of mineral oil can generate droplets of eighty nanoliter size [108]. DMF technologies have been implemented also for the detection of different concentrations of reagents by using gold nanoflowers in a multilayer chip that allows the gradients of the reagents to be deteceted with SERS by Jeon and colleagues [109].

DMF technology is also used as a tool to study the mechanism of viral infection, probing the viral fusion using influenza A to measure the kinetics of fusion of virions with target liposomes. Monitoring the kinetics of fusion was identified by analyzing pH-sensitive fluorescence intensity with the presented method in which virions and liposome were encapsulated within droplets and acidic conditions triggered fusion [110]. 

Single cell analysis of mRNA is a great approach to compare the heterogeneity of biological samples. To improve its performance, microfluidic based DNA-functionalized hydrogel beads which form a matrix to capture mRNA from lysed single cells were developed. To ensure that the mRNA quantification is free of pre-amplification bias, padlock probes and rolling circle amplification (RCA) is used, followed by hybridization with fluorescent probes. The number of transcripts in every cell is assessed by simply counting fluorescent dots inside gel beads [111]. DMF technologies can also provide a tool to simultaneously measure protein expression at a single cell manner. Protein expression can be analyzed from encapsulated individual cells by co-encapsulation with various sensing elements including enzymatic substrates based on color Förster resonance energy transfer (FRET) [112]. 

**Figure 6:**
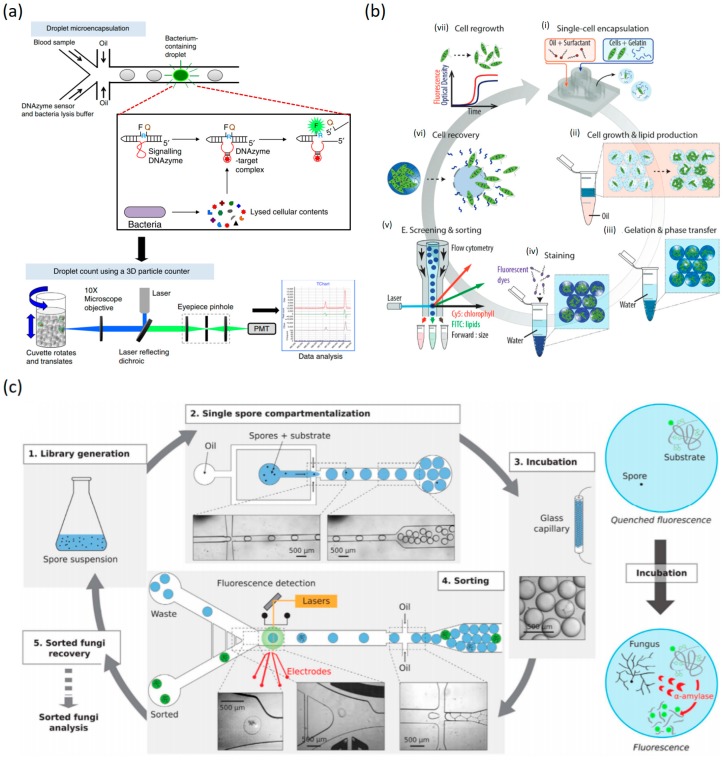
Digital-based microfluidics of cellomics. (**a**) Blood samples and DNAzyme sensors are mixed and then encapsulated in hundreds of millions of micrometer-sized droplets. DNAzyme sensors produce an instantaneous signal in the droplets that contain the bacterium, reproduced with permission from [113], published by Nature, 2014. (**b**) Encapsulation of single microalgal cells within monodisperse gelatin microgel compartments using microfluidic techniques, reproduced with permission from [124], published by John Wiley and Sons, 2018. (**c**) Droplet-based microfluidics screening platform. The screening platform for *Aspergillus niger* was composed of two distinct microfluidic devices, the first one for compartmentalization of spores and the second for sorting them, reproduced with permission from [133], published by Scientific Reports, 2016.

A DNAzyme sensor has also been employed to analyze single bacteria within droplets of unprocessed blood. Encapsulated single *E. coli* was lysed with lysozyme and the intracellular target cleaved the DNAzyme sensor which triggers fluorescence. Then, fluorescent droplets are quantified by using the 3D fluorescent particle counter, allowing the single-droplet detection in a few milliliters of unfluorescent droplets (Figure 6a) [113]. Ng and colleagues demonstrated a multiplexed assay for simultaneous monitoring of six different protease activities from individual cells in a high throughput manner (≈100 cells per experimental run) [112]. Cell signaling is one of the most important processes for both intracellular and extracellular communications to coordinate cellular functions. Currently, much of our knowledge about cell signaling comes from the information obtained from bulk experiments using traditional population-average approaches such as Western blotting assays [114]. While these approaches have been useful to understand intracellular mechanisms, understanding of intracellular phenomena at a single-cell level is one of the challenges and it will provide key information of molecular mechanism within cells. For example, genetic changes, environmental differences and changes in cellular properties produce phenotypic and functional heterogeneity among cancer cells within the same tumor. Droplet microfluidic technology also recently emerged as powerful tool that allows for a high-throughput single cell kinase signaling assay with exceptional sensitivity [115].

DMF technology has been also implemented for the analysis of protoplasts to quantify the levels of chlorophyll and GFP. This application of DMF technology on plant cells analysis can open the opportunity for on-chip fluorescence-based selection of individual plant cells that can be used in targeted regeneration. Combining DMF technology with RNA libraries and gene editing tools like CRISPR-cas9 endonuclease can also improve the academic and industrial research in plant biotechnology [116].

### 5.2. Single Cell Sorting

Fluorescence activated cell sorting (FACS) is one of the most popular technologies for single cell based biological experiment because it can be used to sort single cells according to their size and fluorescence intensity. However, some issues are raised due to their size, cost and complexity for users which makes it difficult to use frequently. Recently DMF technology has provided an alternative approach for single cell sorting which can be potentially used as a cheaper and easier method. 

The analysis and sorting of single mammalian cells is one of the most common procedures in molecular biology research. Cells can be manipulated and the sorting might be divided into active when an external force like magnetic, acoustic or electrical are used for the sorting [117] or passive sorting if it relies only on the interaction with the fluid properties [118]. Cell sorting has been improved since microfluidic technologies were applied to cellomics and various approaches can be used depending on the natural differences of tissue or organ. DMF-based cell sorting approaches include complex steps such as single cell encapsulation, analyzing fluorescence intensity at a single droplet sensitivity within microchannel and intelligent sorting. DMF technologies can improve the throughput of the sorting and analysis but also can give solutions for cell culture, like the use of hydrogel microcapsules that will be a biocompatible niche for three-dimensional in vitro cell culture, where morphology, size and other biochemical properties can be tuned [119] and base the detection in image recognition [120]. Among mammalian cells various types can be detected and sorted like T-cells [121]. Circulating tumor cells are difficult to detect and measure. To improve the detection, new methods based on the Warburg effect had been developed, measuring the levels of lactate and pH on previously encapsulated A549 lung cancer cells, which will not be altered in a blood sample but by reducing the experiment to the picoliter/nanoliter scale can be detected with a pH dye. In clinical assays more experiments are needed to assure that the cells detected are indeed cancer cells [122]. Abate and co-workers recently introduced DMF-based single cell sorting and sorted cells were arrayed for the cultivation. This approach provides a potential tool to construct a large number of relevant single-cellular and multicellular assays [123]. Basically, all kind of cells can be sorted by droplet-microfluidic techniques like bacteria, viruses, and fungi. Even producing single-cell-derived microalgal clones in monodisperse gelatin microgel compartments, growing them and sorting them by staining specific metabolites in microgel beads is another possible application of droplet microfluidics that might be useful in the industry of bioproducts (Figure 6b) [124]. Some sorting methods like PCR-activated cell sorting have been developed where the fluorescence is activated by a TaqMan probe, allowing any kind of cell to be sorted based on its genome or to some specific mutation [125]. The cell type composition can be determined by single-nucleus RNA-sequencing which can analyze transcriptional states in vivo and cell fixation methods contribute to a better resolution [126,127].

DMF high-throughput screening combined with mutagenesis strategies allowed the identification of vitamin-secreting lactic acid bacteria, using roseoflavin-resistant mutant of *L. lactis* strain MG1363, JC017, which secreted low levels of riboflavin. Using fluorescence-activated droplet sorting, it has been shown that various mutants that secreted riboflavin more efficiently than JC017 were readily isolated from the mutagenesis library, then candidates with as few as 1.6 mutations per million base pairs (Mbp) were isolated with a highly efficient screening [128]. Other methods for single-cell isolation have been used for genome analysis and cultivation of various microorganisms [129]. The identification of a single type of bacteria and its functionality, by combining FACS, HLPC and mass spectrometry can be another application [130]. A DMF platform has been developed for studying the interaction of the *Bacillus* strains present in the oral microbial community of the Siberian bear, which shows that profiling the activity of microbiota by DMF technology can improve how to quantify the influences on the microbiome as well as for discovering new antibiotics [20].

Virus can be sorted by DMF devices like Lance and colleagues who introduced a method to isolate a virus from heterogeneous populations that provides advantages compared to FACS. PCR-associated virus sorting needs only 100 bp to generate TaqMan probes. This method also can detect virus present within host, bacteria and eukaryotic cells, encapsulating host cells with the virus and then the lysate undergoing PCR, producing a positive signal if the virus is present in the host [131]. Another application is the sorting of HIV-1 particles in a single-virus DMF platform, with an efficiency higher than 99%, identifying, sorting and analyzing the neutralizing epitopes that might be antigenic features for HIV vaccine candidates [132].

Sorting of fungi is usually high cost and low throughput, however, DMF technologies were used for a screening based in an enzymatic assay reducing the off-chip manipulation of fungi and the time and cost of the procedure by adjusting to nanoliter-volume droplets. Combining microfluidic and the robotic microtiter-plate High-throughput screening (HTS) the group of Thomas Beneyton, compartmentalized in droplets single fungal spores together with a fluorogenic α-amylase substrate. Enzyme secretion was coupled with the fluorogenic α-amylase reaction, and the substrate was completely consumed in many droplets. The throughput of microfluidic HTS allows the screening of highly mutated libraries, which are composed mainly of inactive clones, but also the higher mutation rate increases the chance of hitting target genes which lead to a high probability of finding beneficial mutations, compartmentalizing the single spores and performing amylase. Recently, a DMF-based approach has been used to study amylase that was produced by the fungi through hydrolyzing the starch backbone and unquenching the fluorophores inducing fluorescence (Figure 6c) [133]. Fungi sorting can be useful in biotechnological industry processes like the pharmaceutical or food industries, and droplet microfluidics can be a useful tool for monitoring the enzyme production by fungi [134]. The DMF approach also can provide a method for single cell array at a high throughput manner. Cole and colleagues presented a printed DMF that provides a programmable and robust technology to build arrays of defined cell and reagents combinations and allows the integration of multiple measurement modalities together in a single assay [123]. 

### 5.3. Single Cell Engineering

Last years, modification and even synthesis of complete cells has been a major tool for research in which reducing the scale allowed better approaches and results. DMF technology has been proved as a very effective tool for the development of new cell engineering techniques. Among them CRISPR is probably one of the most exciting topics in molecular biology that has emerged these last few years; combined with droplet microfluidics tools, the delivery of CRISPR has been used for gene therapy to knock down the TP53BP1 gene from K562 cells [135,136]. DMF technology can also improve plasmid transfection to single cells; monodisperse lipoplexes are included with single cells in the droplets using plasmid pcDNA3 for the transfection which improved efficiency from 5% to 50% [135] as well as lentivectors [137].

A very interesting application of DMF technology is the construction of synthetic cells. The assembly of unilamellar vesicles and compartmentalization by pico-injection, merging lipid vesicles and copolymers-stabilized droplets for generating stable cell-like compartments, have been called droplet-stabilized giant unilamellar vesicles (dsGUVs) [138]. A double emulsion precursor form has been utilized to improve the storage limitations of all kinds of cell-sized lipid vesicles (CLVs). The therapeutic potential of these lipid vesicles has been demonstrated utilizing them to present transmembrane proteins like neuroligin-2 to pancreatic β-cells, stimulating insulin secretion with the formation of cell–cell synapses [139].

DMF technology can produce monodisperse droplets controling the size precisely and tailoring the internal structure to incorporate different cells in designed locations. For example, Chen and colleagues reported a new method for the development of organ-on-a-chip for the first time producing highly monodisperse structures that enable multiple types of cells to arrange them to create an artificial liver in a drop [140].

## 6. Droplet-based Microfluidics for the Field Applications

Recently, biochemist and molecular biologist have particular interest in accomplishing the challenge of providing useful technologies which can be used in real life problems such as biomedical diagnosis and among other fields like drug screening and development. Probably one of the most used tools in these diagnostic field is PCR. DMF technologies update this procedure like we have already discussed in this paper. Digital PCR can provide a robustness and convenience which can mitigate the effects of PCR inhibitors in diluted samples, regardless of the form of template (i.e., cells or genomic DNA) for forensic short tandem repeat (STR) typing at the single-cell/molecule level [141].

### 6.1. Diagnosis

Among all the possible applications of DMF technology, clinical diagnosis is one of the most promising fields which can benefit from this technology by increasing the throughput and reducing the time needed to obtain the results which is of paramount importance when we turn to diagnosis.

The detection of RNA and DNA levels in DMF devices is a powerful tool for diagnosis of various diseases like infections or cancer; several examples have been shown such as controlling the levels of overexpression of genes in various cell lines [142].

Sensing biomarkers is another application that has improved these past few years, showing various examples like detecting the levels of glucose which can be sensed more precisely with DMF electrochemical sensors using platinum-black electrode generated by micro deposition of Pt nanoparticles on bare Pt microelectrode. The levels of glucose oxidase can be detected up to 43.5 mM offering possibilities for developing droplet-based highly sensitive and low consumption sensors [99]. Multiplex detection of different biochemical markers has been obtained by the integration of microvalves for the control of droplet generation. This platform utilizes a colorimetric and fluorescence-based assay to detect at same time biomarkers like lactate dehydrogenase (LDH), bile acid and glucose in a device connected to a cell culture system which contains hepatocytes. It makes it possible to integrate multiplexed sensing in organ-on-a-chip devices [143]. 

In the past decade, DMF technology has been integrated in devices already commercialized like the Bio-Rad digital droplet PCR system which has been used for multiplex DNA targets quantification [144]. Raindance technology® developed a digital PCR based device that can interrogate mutations in 50 oncogenes, as well as tumor suppressors and drug resistance markers, generating 8 million picodroplet reactions per well. Velox Biosystems also commercialized IC3D, a system that provides a rapid (less than 3 h) and highly sensitive detection technology for a specific type of bacteria in unprocessed blood. Quanterix® designed a fully automated immunoassay system for multiplexed and customized assay capability and the Mission bio Tapestri commercialized single cell-encapsulation technology that divides thousands of cells into individual droplets, enabling thousands of individual reactions simultaneously, from cell lysis and protease digestion to access to DNA for further amplification.

### 6.2. Drug Screening

Drug screening is one of the applications which can benefit greatly from DMF technologies allowing the performance of high-throughput screenings in a shorter time. There are various DMF-based approaches have been developed and widely applied for drug screening. Recently for the screening of more than 56 conditions and 20 replicates on different pancreatic cell lines, regulating the flow of the different 16 syringes to produce different combinations [145]. In their approach, live tumor cells were used for drug screening, without an ex vivo cultivation step, that provides higher sensitivity profiles against drug candidates. With this DMF-based approach, the entire work flow can be completed within 48 h, with a cost of $150 each assay per patient. It will provide an emerging tool for rapid determination of optimal personalized medication for cancer patients. The on-demand trapping and releasing of droplets controls the pressure in the channel networks which can be applied to the screening [146]. Another trapping array device for the study of cell-penetrating peptides (CPP) in population-based analysis has been developed allowing single-cell fluorescence imaging of CPP [147]. A DMF device based on PDMS was used for conducting drug screening with suspended and attached cancer cells screening. A similar approach was utilized in a drug susceptibility test for MCF-7 cells where even 80,000 cells could be sorted [148]. Even the release of drugs controlling the surface texture of the polymer [149]. DMF has been utilized also for the development of diffuse large B cell lymphoma (DLBCL) immunogenic spheroids where it can determine the cell adhesion, aggregation, proliferation and interaction between cancer cells, fibroblasts and lymphocytes providing a high-throughput that facilitates the study of the drug effects over the whole immunogenic tumor microenvironment [150]. 

## 7. Conclusion

Recent advances in DMF technologies for biochemistry and molecular biology have been introduced and discussed in this review. It can be seen that biochemistry and molecular biology have found in DMF a technology for improving the sensitivity, accuracy, speed and throughput of experiments (Table 1). 

The advances in sequencing and detection, and protein and single cell detection in DMF can be a great boost for basic research and clinical purposes. However, more advances in the development of devices for point of care testing has to be achieved, integrating DMF devices in complete instruments will be one of the challenges for the next few years in the field [154] (Table 2). DMF also became a powerful tool for bioengineering at a single-cell level by combining with classic techniques like plasmid transfection to recently-developed technologies such as CRISPR [135]. These applications are the proof of the many possibilities that DMF gives not only for research in academic laboratories, but also opens opportunities to develop new techniques in diagnosis and also for the personalized medication. There are already various DMF-based powerful tools, but it will be expected that MDF technologies will be continually developed to solve the hurdles faced by biochemists and molecular biologists. 

## Figures and Tables

**Table 1:** Comparison of throughput and sensitivity of droplet-based microfluidics and selected conventional approaches.

Selected Applications	Throughput		Limit of Detection	
	Conventional Methods	DMF	Conventional Methods	DMF
Single-cell DNA sequencing	384 cells/assay [136]	50,000 cells/run [12]	10–20 kb templates [13] (Minimum size of DNA that can be analyzed)	3 kb can be analyzed [13] (Minimum size of DNA that can be analyzed)
PCR	1 reaction/20 µL [21]	2 million reactions/nL [21]	One mutation in 20,000 wild-type of background DNA [151]	One mutation in 5 million wild-type of background DNA [151]
ELISA	96 or 384 reaction/assay [74]	500 reaction/assay [74]	0.1–0.2 µg/mL [74]	0.01 µg/mL [74]
Single-cell sorting	1536 cells/assay [152]	100,000 droplets/s [113]	80 cells/mL [153]	10 cell/mL [113]
Directed evolution	73,000 reactions/day [81]	1 × 10^8^ reactions/day [81]	NA	NA
Drug screening	3328 cells/screening [148]	80,000 cells/screening [148]	1 × 10^5^ cells [148] (Minimum requirement of cells for screening)	10–100 cells [148] (Minimum requirement of cells for screening)

**Table 2:** Advantages and disadvantages of droplet-based microfluidics for selected applications.

Selected Applications	Droplet Microfluidics	
	Advantages	Limitations
Single-cell sequencing	Rapid (21,000 cells/h) [47]	Requires specific microfluidic device and instrument for droplet sorting
PCR	Sensitivity (1–10^11^ copies per reaction) and accurate (<2% standard deviation) [22]	
ELISA	Reduction of sample and reagent volume, decrease operation time, no need for purification steps [74]	
Single-cell sorting	Reduced amount of reagent (10 cells/mL), faster sorting time (100,000 droplets/s) [113]	
Directed evolution	Reduced amount of reagent (<6 pL) and Increased efficiency (1 × 10^8^ reactions/day) [81]	
Drug screening	Increased the number of drugs and cell samples tested (80,000 cells/screening), low reagent consumption (<3 mL) [145,148]	
